# Factorized Computation: What the Neocortex Can Tell Us About the Future of Computing

**DOI:** 10.3389/fncom.2018.00054

**Published:** 2018-07-31

**Authors:** Peter U. Diehl, Julien Martel, Jakob Buhmann, Matthew Cook

**Affiliations:** Institute of Neuroinformatics, The University of Zurich and ETH Zurich, Zurich, Switzerland

**Keywords:** factorized computation, relational networks, unified brain theory, non-sequential, parallelization

## 1. Introduction

In ancient Greece our brains were presumed to be mainly important for cooling our bodies. When humanity started to understand that our brains are important for thinking, the way it would be explained was with water pump systems as this was one of the most sophisticated models at the time. In the nineteenth century, when we started to utilize electricity it became apparent that our brains also use electrical signals. Then, in the twentieth century, we defined algorithms, improved electrical engineering and invented the computer. Those inventions prevail as some of the most common comparisons of how our brains might work.

When taking a step back and comparing what we know from electrophysiology, anatomy, psychology, and medicine to current computational models of the neocortex, it becomes apparent that our traditional definition of an algorithm and of what it means to “compute” needs to be adjusted to be more applicable to the neocortex. More specifically, the traditional conversion from “input” to “output” is not as well defined when considering brain areas representing different aspects of the same scene. Consider for example reading this paper: while the input is quite clearly visual, it is not obvious what the desired output is besides maybe turning to the next page, but this should not be the goal in itself. Instead, the more interesting aspect is the change of state in different areas of the brain and the corresponding changes in states of neurons. There are many types of models that have the interaction of modules as the central aspect. Among those are:

- Belief propagation
- Dynamic fields
- Relational networks
- Interaction of brain areas in many psychological models
- Basically all models describing ongoing interactions between neurons, e.g., STDP

They all use what we will refer to as “Factorized Computation.” Factorized Computation describes a common framework for distributed processing mechanisms and systems. The term Factorized Computation refers to how a problem gets factorized (decomposed) into smaller sub-problems such that many nodes are working together^1^. Typically, problems are broken down into a large set of small relations, whose composition represents the problem to be addressed.

This decomposition differs from the conventional approach of breaking a problem down into a sequence of subproblems, in that in Factorized Computation the subproblems are interdependent, and they are solved jointly. There is no order for solving the subproblems, just like we don't solve equations one by one–they must be solved together.

Just as functions are defined in terms of other functions or procedures (down to built-in ones), and are chained into sequences in order to transform the input into the output, relations can be defined in terms of other relations (down to built-in ones), and can be arranged automatically into networks of variable nodes and relation nodes, with a connection wherever a variable is involved in a relation.

In this article our goal is to outline how finding and using a unifying framework for the aforementioned models can benefit our understanding of the neocortex, and how the process of understanding the neocortex can inspire new models and build a foundation for the next generation of hardware.

### 1.1. The “individual-thread” approach

The first computing architectures ever made implemented an idea that seems very natural to us: A task can be performed by dividing it into several sub-tasks to be carried out in a sequential order. As humans, this idea is natural to us since this is how we typically approach tasks in the world around us. We do one sub-task at a time, whether for physical tasks or paper-and-pencil tasks. Even when speaking about thought processes, we refer to “a train of thoughts,” reflecting our view that thinking involves a sequence of thoughts. Instructions are often arranged for clarity in numbered steps, making their sequential nature explicit. We may be instructed to skip to another point in the sequence, but we are practically never instructed to carry out two or more such sequences simultaneously.

### 1.2. Is it individual threads all the way down?

High level routines (in code) or procedures (for people) are often specified as a sequence of lower level routines, which themselves also use the individual-thread approach, i.e., they call a sequence of even lower level routines, and so on. Is this, then, what computation is, at heart? Is this the general method used by objects that compute?

Even though it might seem, as seen from the outside, that thinking is a single threaded-activity, or that the internal workings of a CPU are single-threaded, in fact these systems are strongly parallel on the inside. Looking inside our brain, we see it has activity going on simultaneously in many different areas, collectively contributing to achieving one task, but with each area working asynchronously under its own control. Similarly, a modern processor has many activities going on simultaneously within a busy core, to aid in the processing of the main thread. So we see that below the level of the processor (whether CPU core or human), operations are highly parallelized.

At higher levels, operations are again highly parallel. For example the employees and departments of an organization all work simultaneously, whether working together on shared goals or separately on distinct goals. So it is mainly at the level of the individual that we see a difficulty with parallelization. Similarly, the multiple cores of a CPU or GPU can also all work simultaneously, barely aware of each other. It is only at the single core level that operations take on a purely sequential nature. This is not a coincidence, since after all, the theoretical understanding underlying a core's operation (and indeed, underlying much of theoretical computer science) is historically based on modeling what an individual person does.

This individual-thread approach to computing is largely a result of how we have trained ourselves to think and reason about computation. The idea of “computing” meaning the “execution of a program,” and a “program” as a “sequence of operations,” is very deeply ingrained in us, to the point that we are quick to see any computational process as the execution of a program in some sense. If there are other frameworks that would be better suited for understanding a given computation, we often don't even notice them, because we are so quick to understand distributed computations in terms of the framework we are used to: a large number of parallel, individual threads, each following a sequence of instructions.

## 2. A recurring theme: factorized computation

Many of the formalisms that have been proposed for specific types of parallel computations share an underlying theme in how they are structured and many separate developments in different fields have repeatedly led to a similar style of distributed calculation, which we refer to collectively as Factorized Computation. Specific instances include Bayesian Brain theories (Knill and Pouget, 2004), dynamic fields (Wilimzig and Schöner, 2005), and relational networks (Diehl and Cook, 2016). Also, neuroanatomy tells us that connections between areas are almost always reciprocal/bidirectional (not on a per-neuron level but on a per-area level) (Felleman and Van Essen, 1991). This is in stark contrast to traditional feed-forward processing models and since reciprocal connections double the required resources, we would expect there to be a clear need for these connections in brain models.

Factorized Computation is closely related to relational processing, an umbrella term covering many specific styles of setting up computations as some sort of relational network. Such a network consists of a set of variables, together with a set of relations between those variables, such as clauses or equations. The relations in the network behave as active elements rather than being used or inspected by a separate entity tasked with satisfying them. Each relation pays attention to the variables it relates, communicating with any other relations involving the same variables. A relation listens to its neighbors' opinions about its variables, and by considering these opinions in the light of its own mathematical relation, it forms or updates its own opinion about its variables, which it then shares with its neighbors (an example is given below).

For Factorized Computation, the approach to “programming” is to examine the concepts with which we understand the quantity or behavior to be computed, and to directly model those quantities and their relationships, instead of modeling the sequence of steps you would take if doing the computation yourself. This is like writing down the equations that constrain the answer, without specifying how to solve the equations.

This approach is almost the opposite of the standard parallelization of the “embarrassingly parallel” parts of a problem. The parts of a Factorized Computation are not independent, but are continuously communicating with each other in order to approach a solution.

### 2.1. A simple example

As an example, a node in a network might represent the relation *A* + *B* = *C*. This node would be connected to other nodes involving these variables; for example, it might have a neighbor representing *C* + *D* = *E*−*F*, and these two nodes would send each other information about the value of *C*. If the *A* + *B* = *C* node hears from its neighbors that *A* is around 2, *B* is around 3, and *C* is around 4, then it might in turn tell its neighbors that *A* is probably a little less than 2, *B* is probably a little less than 3, and *C* is probably a little more than 4. Or, with a different formalism for how to interpret values, if it hears that *A* = 2 and *B* ≥ 3, it might tell its neighbors that *C* ≥ 5.

Other formalisms might send samples from distributions representing the current posterior for the variable, or send the set of remaining possible values for the variable as inconsistent values get ruled out, or simply send the current best estimate of the variable's value. There are many such formalisms (Hinton and Sejnowski, 1986; Rumelhart and McClelland, 1987; Wegener, 1987; Shapiro, 1989; Dechter, 2003; Diehl and Cook, 2016), but the intuition behind creating the network is the same in each case: The variables are all the quantities you think about or compute when thinking about the problem, and the relations encode the way in which these variables are related to each other.

As we can see, the calculation done at a single node is extremely simple. It is not a powerful equation solver—it is more like a neuron, capable of only a fixed operation. Just like neurons, the power of these systems arises from having many nodes working together.

## 3. Outlook

### 3.1. Where can we go from here?

While it is nice to observe that many existing algorithms are examples of Factorized Computation, and that there are surely many more algorithms of this form waiting to be discovered, is there anything in particular that really needs to be done, or should we just sit back and enjoy the show?

For comparison, if we look at the history of one particular form of Factorized Computation, namely belief propagation in factor graphs, we see that this formalism was developed multiple times over many years by many researchers. First developed in periodic forms by statistical physicists (Bethe, 1935), it was developed again 30 years later in various linear forms for decoding noisy signals in the field of communications (Viterbi, 1967), and 20 years later it was developed yet again to reason about causal relationships in the field of artificial intelligence (Pearl, 1988). After another decade, in retrospect (Kschischang et al., 2001) it became clear that a lot of effort could have been saved if the general form of these algorithms had been explicitly understood, rather than being worked out repeatedly and independently for what now appear to be various special cases of a general theme.

### 3.2. Factorized computation: a direction

There are many existing examples of systems within neuroscience which can be understood as Factorized Computation, such as belief propagation (Knill and Pouget, 2004), dynamic fields (Wilimzig and Schöner, 2005), relational networks (Diehl and Cook, 2016), or the interaction of brain areas in many psychological models. As we try to gain a more complete and unified understanding of this framework, a number of directions already present themselves. One direction that has the potential to greatly increase the range of applicability of Factorized Computation is improving the automated learning of latent variables and the relations between them. This direction includes how relations in deep hidden layers can be trained, as well as how other encodings such as temporal sequences can be usefully learned and used. We could even ask how the network of variables and relations could itself arise from a homogeneous sheet of computing elements, much as the cortical sheet of the brain self-organizes into areas that specialize in specific tasks.

Another direction where Factorized Computation is well-suited to applications is in understanding and re-engineering vision systems, where it is possible to use a large relational network to decompose sensory input into multiple physically-meaningful modalities. For example, from a retina or a dynamic vision sensor which reports only changes in light intensity without absolute brightness information, a network can infer the optic flow, motion, and even the missing brightness information (Martel et al., 2015). Such networks are in principle capable of integrating input from more types of sensors, such as vestibular system (accelerometers), auditory system and so on. The Factorized Computation framework provides a very natural and general method for the problem of sensor fusion, where the more sensors you include, even of completely different types, the more accurate the entire system will be. Even distinct algorithms could be combined, providing “algorithm fusion” for example to combine multiple methods of computing optic flow. Constructing and optimizing such brain-like frameworks and systems will allow us to better understand how our brains function and why they developed the way they did.

## Author contributions

All authors listed have made a substantial, direct and intellectual contribution to the work, and approved it for publication.

### Conflict of interest statement

The authors declare that the research was conducted in the absence of any commercial or financial relationships that could be construed as a potential conflict of interest.

## Footnotes

^1^Some formalisms express the entire problem as a product or conjunction of many terms, and in this case the terms are factors in a multiplicative sense. Other formalisms are not based on multiplication, but we still speak of the relations as factors that influence the desired computation, and we speak of a computation as having been factorized when it has been broken down into the relations that define it.

## References

[B1] Bethe H. A. (1935). Statistical theory of superlattices. Proc. R. Soc. Lond. A Math. Phys. Eng. Sci. 150, 552–575.

[B2] Dechter R. (2003). Constraint Processing. San Francisco, CA: Morgan Kaufmann.

[B3] Diehl P. U. Cook M. (2016). Learning and inferring relations in cortical networks. arXiv:1608.08267.

[B4] Felleman D. J. Van Essen D. C. (1991). Distributed hierarchical processing in the primate cerebral cortex. Cereb. Cortex 1, 1–47. 1822724 10.1093/cercor/1.1.1-a

[B5] Hinton G. E. Sejnowski T. J. (1986). Learning and relearning in Boltzmann machines, in Parallel Distributed Processing: Explorations in the Microstructure of Cognition, Vol. 1, eds Rumelhart D. E. McClelland J. L. (Cambridge, MA: MIT Press), 282–317.

[B6] Knill D. C. Pouget A. (2004). The Bayesian brain: the role of uncertainty in neural coding and computation. Trends Neurosci. 27, 712–719. 10.1016/j.tins.2004.10.007 15541511

[B7] Kschischang F. R. Frey B. J. Loeliger H.-A. (2001). Factor graphs and the sum-product algorithm. IEEE Trans. Inform. Theor. 47, 498–519. 10.1109/18.910572

[B8] Martel J. N. P. Chau M. Dudek P. Cook M. (2015). Toward joint approximate inference of visual quantities on cellular processor arrays, in 2015 IEEE International Symposium on Circuits and Systems (ISCAS) (Lisbon), 2061–2064.

[B9] Pearl J. (1988). Probabilistic Reasoning in Intelligent Systems: Networks of Plausible Inference. San Francisco, CA: Morgan Kaufmann.

[B10] Rumelhart D. E. McClelland J. L. (1987). Parallel Distributed Processing, Vol. 1 Cambridge, MA: MIT Press.

[B11] Shapiro E. (1989). The family of concurrent logic programming languages. ACM Comput. Surv. 21, 413–510.

[B12] Viterbi A. J. (1967). Error bounds for convolutional codes and an asymptotically optimum decoding algorithm. IEEE Trans. Inform. Theor. 13, 260–269.

[B13] Wegener I. (1987). The Complexity of Boolean Functions. New York, NY: John Wiley & Sons, Inc.

[B14] Wilimzig C. Schöner G. (2005). The emergence of stimulus-response associations from neural activation fields: dynamic field theory, in Proceedings of the 27th Annual Conference of the Cognitive Science Society (Stresa).

